# What Can We Learn from the Scalp Hair’s Trace Element Content? The Relationship with the Advancement of Coronary Artery Disease

**DOI:** 10.3390/jcm13175260

**Published:** 2024-09-05

**Authors:** Tomasz Urbanowicz, Anetta Hanć, Julia Frąckowiak, Maksymilian Białasik-Misiorny, Zofia Radek, Marta Krama, Krzysztof J. Filipiak, Aleksandra Krasińska-Płachta, Sylwia Iwańczyk, Mariusz Kowalewski, Andrzej Tykarski, Marek Jemielity

**Affiliations:** 1 Cardiac Surgery and Transplantology Department, Poznan University of Medical Sciences, 61-701 Poznan, Poland; 2 Thoracic Research Centre, Collegium Medicum Nicolaus Copernicus University, Innovative Medical Forum, 85-094 Bydgoszcz, Poland; 3 Department of Trace Analysis, Faculty of Chemistry, Adam Mickiewicz University, 61-614 Poznan, Poland; 4 Poznan University of Medical Sciences, 61-701 Poznan, Poland; 5 Department of Hypertensiology, Angiology and Internal Medicine, Poznan University of Medical Sciences, 61-701 Poznan, Poland; 6 Institute of Clinical Science, Maria Sklodowska-Curie Medical Academy, 00-136 Warsaw, Poland; 7 Department of Ophthalmology, Poznan University of Medical Sciences, 61-107 Poznan, Poland; 8 1st Cardiology Department, Poznan University of Medical Sciences, 61-107 Poznan, Poland; 9 Department of Cardiac Surgery and Transplantology, National Medical Institute of the Ministry of Interior and Administration, 02-507 Warsaw, Poland; 10 Cardio-Thoracic Surgery Department, Heart and Vascular Center, Maastricht University Medical Center (MUMC), Cardiovascular Research Center Maastricht (CARIM), 6229 Maastricht, The Netherlands

**Keywords:** trace elements, coronary disease, scalp hair, atherosclerosis, minerals

## Abstract

**Background**: Multiple risk factors for coronary artery disease have been identified without answering one of the leading questions related to the extent of the involvement of the epicardial arteries. Trace elements are involved in various stages of atherosclerotic lesion formation and may play a significant role in the advancement of coronary artery disease. **Methods**: A total of 157 (92 (59%) men and 65 (41%) women) patients with a median age of 71 (65–75) years presenting with chronic coronary syndrome were enrolled in the prospective single-center analysis. The coronary angiography results were compared with the concentration of trace elements in scalp hair. **Results**: Through Spearman analysis, a positive correlation between the number of diseased coronary arteries and hair trace element concentration was found for sodium (r = 0.198, *p* = 0.013), vanadium (r = 0.164, *p* = 0.040), chromium (r = 0.242, *p* = 0.002), and nickel (r = 0.176, *p* = 0.026). A negative relationship was noted between magnesium (r = −0.237, *p* = 0.003) and calcium (r = −0.217, *p* = 0.007) and the extent of epicardial lesions. **Conclusions**: Scalp hair trace element analysis indicates the possible modulatory role of trace elements in advancing coronary artery disease. Since a significant correlation with one- and two-vessel but not with three-vessel disease was noted, it might be considered an “all or nothing” phenomenon. A positive correlation between the number of diseased coronary arteries and sodium, vanadium, chromium, and nickel and an inverse correlation with magnesium and calcium were noted. The presented analysis is hypothesis-generating, and further studies are necessary to corroborate the results from a clinical perspective.

## 1. Introduction

Coronary artery disease represents one of the greatest threats to human health due to its high morbidity rates in the current population [1]. Atherosclerotic plaque formation results from chronic, lipid-driven inflammatory processes involving endothelial cell activation, monocyte infiltration, and foam cell generation [2]. These complex processes are triggered by multiple factors. Endothelial dysfunction is claimed to be a primary determinant of atherosclerosis initiation, including exposure to shear stress (WSS) and non-linear blood flow [3,4].

The role of inflammation in coronary lesion formation and progression is complex, and recent studies confirmed the association between the severity of coronary artery disease and reactive markers from peripheral blood, such as cytokines (IL-6, IL-1β, TNF-α) [5]. Oxidative stress affects the production of vasoactive and proinflammatory prostaglandins, leading to endothelial function disturbances, resulting in increased endothelial permeability, platelet aggregation, and leukocyte adhesion [6]. In animal studies, the role of macrophages in postinfarction healing processes was also presented [7], pointing out their role in lesion progression and heart recovery. As immunological activation may result in lesion formation [6,7], even simple inflammatory markers may possess prognostic value in multivessel coronary disease [8].

Essential metals are believed to be involved in atherosclerotic lesion formation as protein cofactors involved in redox reactions, cell differentiation, and gene expression. Significant variability exists in trace elements’ involvement in atherosclerosis insurgence and plaque evolution. Their multi-faceted and complex role in pathogenesis is still a matter of investigation [9]. Trace elements involved in atherosclerosis formation and progression can be divided into those related to heavy metal exposure, components involved as co-factors in pathobiological processes, and reactions triggered by a concentration imbalance in the body [10,11].

The extent of atherosclerotic plaque in the coronary lumen and the location of coronary artery disease (CAD) exhibit not only diverse clinical presentations but also diverse prognoses. It is claimed that anatomic followed by hemodynamic characteristics may play a role in lesion location. Among the demographical and clinical factors, age and sex are related to coronary lesion topography [12]. The increased risk for atherosclerotic lesions related to heavy metal body accumulation in certain groups, including obese patients, was presented by Skalny et al. [13].

This study aimed to measure the trace element concentrations in scalp hair samples in relation to coronary disease advancement.

## 2. Materials and Methods

Patients:

A total of 157 (92 (59%) men and 65 (41%) women) patients with a median age of 71 (65–75) years were enrolled in the prospective single-center analysis in this study. All patients were admitted to the cardiology department presenting with chronic coronary syndrome and referred by their cardiologist for coronary angiography. Overall, 93 (59%) presented with anginal symptoms upon exertion and 64 (41%) reported shortness of breath who were classified as median CCS (Canadian Cardiovascular Society) class 2.0 (1.8–2.2). Patients with acute coronary syndromes or previous percutaneous or surgical coronary interventions were excluded from the analysis. The analysis did not include patients living in highly industrialized areas or exposed to heavy-metal-rich environments. Potential dietary effects were ruled out. The patients’ characteristics are presented in Table 1.

### 2.1. Hairs Scalp Methodology

Hair measuring 2–3 cm in length was trimmed from the occipital region of the head, near the scalp, without any permanent wave or dye treatment. The gathered hair samples were washed and dried following the procedure outlined in [14]. Dry hair samples weighing 150–200 mg underwent mineralization in the DigiTube system (SCP et al.). The mineralization process involved using 4 mL of 65% nitric acid (Suprapure et al.) and 1 mL of 30% hydrogen peroxide (Supelco, Merc, Germany). These prepared samples were then heated at 150 °C for 4 h. After cooling to room temperature, the samples were diluted 100-fold with Milli-Q water (Millipore Direct Q-3, Merc, Germany). Subsequently, the samples were analyzed for elemental content using the solution nebulization ICP-MS method (Agilent 7700x, Poznan, Poland), following the procedure previously described by Urbanowicz et al. [15]. The validity of the analytical method was assessed by analyzing the certified reference material (CRM), NCS ZC 81002b Human Hair (Beijing, China). Trueness was evaluated by comparing recovery values (%) obtained from the certified reference material, ranging from 94% to 107%.

### 2.2. Statistical Analysis

The normality of the distribution of variables was tested with the Shapiro–Wilk test. The *t*-test, Cochran–Cox test, Mann–Whitney test, and Fisher’s exact test were used where applicable to compare the variables between two groups. Spearman correlation analysis was used to describe the correlation between the variables. Statistical analysis was performed using Statistica 13 by TIBCO. *p* < 0.05 was considered statistically significant.

### 2.3. Bioethics Committee Approval

This study was performed according to the principles of Good Clinical Practice and the Declaration of Helsinki. It was approved by the Local Ethics Committee of the Medical University of Poznan (approval number: 875/22 on 3 November 2022). All patients gave their informed consent for inclusion in this study.

## 3. Results

All patients underwent coronary angiography, which revealed normal angiograms in 74 (47%) patients, while 83 (53%) of patients presented with coronary atherosclerosis located in one (30 (36%) patients), two (13 (16%) patients), and three (40 (48%) patients) epicardial arteries.

There was a relationship noted between clinical factors such as diabetes mellitus (*p* = 0.022) and the number of diseased epicardial arteries, but not regarding hypertension (*p* = 0.109) and dyslipidemia (*p* = 0.329) in the presented group.

The scalp hair samples were collected from consecutive patients referred due to chronic coronary syndrome before angiograms. The estimation of the scalp hair’s trace element content was performed, and these data are presented in Table 2.

Patients presenting with atherosclerotic lesions in coronary angiograms were characterized by statistically significant differences in their scalp hair concentrations of sodium (Na) (*p* = 0.002), magnesium (Mg) (*p* = 0.012), potassium (K) (*p* = 0.002), calcium (Ca) (*p* = 0.007), vanadium (V) (*p* = 0.007), chromium (Cr) (*p* < 0.001), iron (Fe) (*p* = 0.006), and strontium (Sr) (*p* = 0.007).

### Correlations

**Figure 1:**
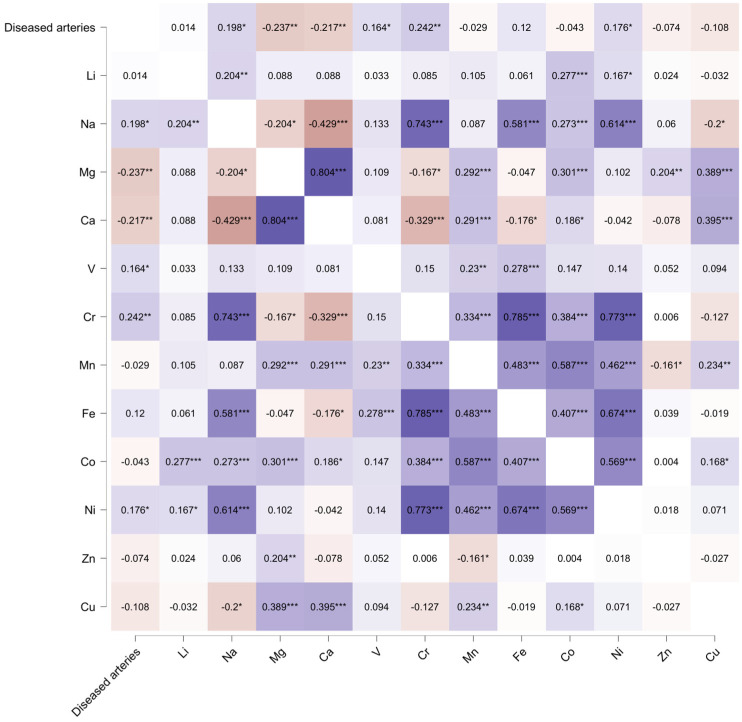
A positive correlation between the number of diseased coronary arteries and the hair trace element concentration was found for sodium (r = 0.198, *p* = 0.013), vanadium (r = 0.164, *p* = 0.040), chromium (r = 0.242, *p* = 0.002), and nickel (r = 0.176, *p* = 0.026). * *p* < 0.050, ** *p* < 0.010, *** *p* < 0.001.

The Spearman test presented a possible correlation between scalp hair trace element concentrations and coronary artery disease risk, including the number of involved arteries (Figure 1).

**Figure 2:**
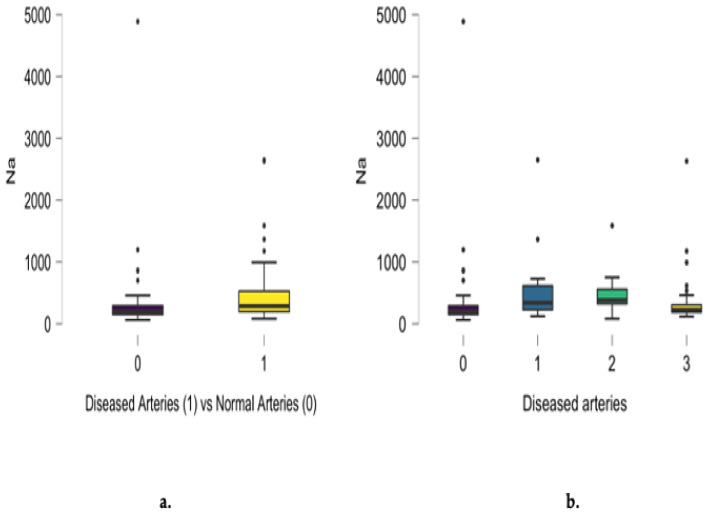
Differences between diseased and normal angiograms regarding scalp hair sodium concentration (mg/kg) (**a**) and the positive correlation between angiographic results and the number of diseased coronary arteries (r = 0.198, *p* = 0.013) (**b**).

Differences between diseased and normal angiograms regarding scalp hair sodium concentration were noted (*p* = 0.002), as presented in Figure 2a. A positive relationship between sodium concentration and the number of involved arteries was noted for single- and two-vessel disease. Non-significant differences in sodium scalp hair concentration also characterized patients presenting with three-vessel disease. Analyzing the entire group, a positive correlation between angiographic results and scalp hair sodium concentration was noted (r = 0.198, *p* = 0.013), as presented in Figure 2b.

**Figure 3:**
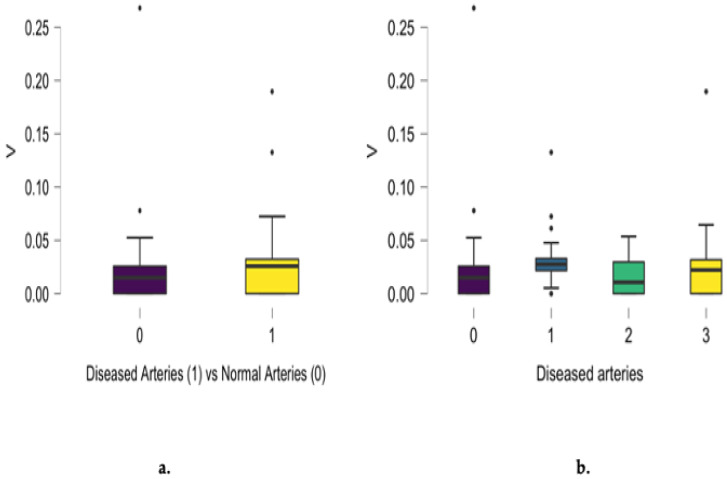
Differences between diseased and normal angiograms regarding scalp hair vanadium concentration (mg/kg) (**a**) and the positive correlation between angiographic results and the number of diseased coronary arteries (r = 0.164, *p* = 0.040) (**b**).

Differences between diseased and normal angiograms regarding scalp hair vanadium concentration were noted (*p* = 0.007), as presented in Figure 3a. A positive relationship between vanadium concentration and the number of involved arteries was noted for single-, two-, and three-vessel disease. The highest differences in vanadium scalp hair concentration were noted between patients presenting with normal angiograms and those with single-vessel disease. Analyzing the entire group, a positive correlation between vanadium concentration and the number of diseased coronary arteries was noted (r = 0.164, *p* = 0.040), as presented in Figure 3b. 

**Figure 4:**
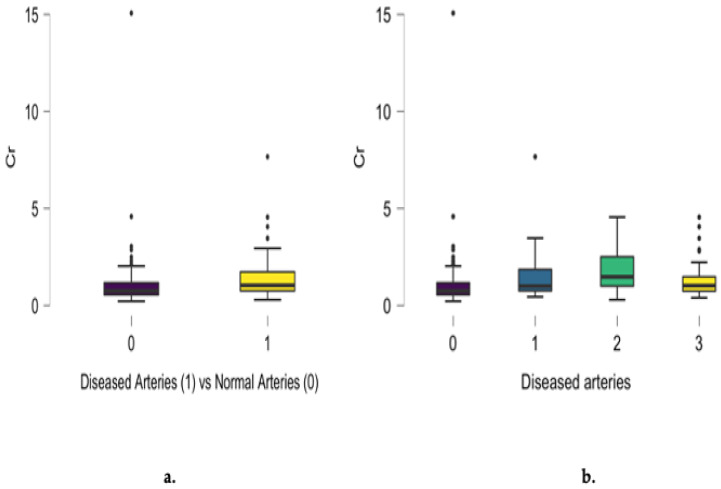
Differences between diseased and normal angiograms regarding scalp hair chromium concentration (mg/kg) (**a**) and the positive correlation between angiographic results and the number of diseased coronary arteries (r = 0.242, *p* = 0.002) (**b**).

Differences between diseased and normal angiograms regarding scalp hair chromium concentration were noted (*p* < 0.001), as presented in Figure 4a. A positive relationship between chromium concentration and the number of involved arteries was noted for single- and two-vessel disease. Non-significant differences in sodium scalp hair concentration also characterized patients presenting with three-vessel disease. Analyzing the entire group, a positive correlation between chromium concentration and the number of diseased coronary arteries was noted (r = 0.242, *p* = 0.002), as presented in Figure 4b. 

**Figure 5:**
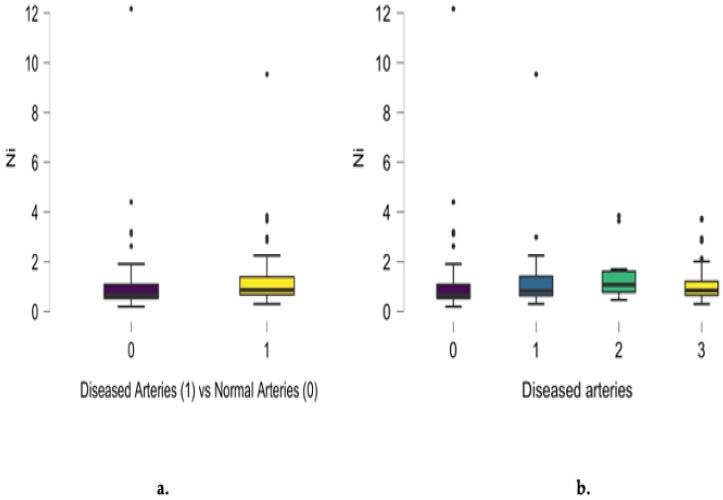
Differences between diseased and normal angiograms regarding scalp hair nickel concentration (mg/kg) (**a**) and the inverse correlation between angiographic results and the number of diseased coronary arteries (r = 0.176, *p* = 0.026) (**b**).

Non-significant differences between diseased and normal angiograms regarding scalp hair nickel concentration were noted (*p* = 0.333), as presented in Figure 3a. A positive relationship between nickel concentration and the number of involved arteries was noted for single- and two-vessel disease. Non-significant differences in nickel scalp hair concentration also characterized patients presenting with three-vessel disease. Analyzing the entire group, a positive correlation between nickel hair concentration and the number of diseased coronary arteries was noted (r = 0.176, *p* = 0.026), as presented in Figure 5b.

**Figure 6:**
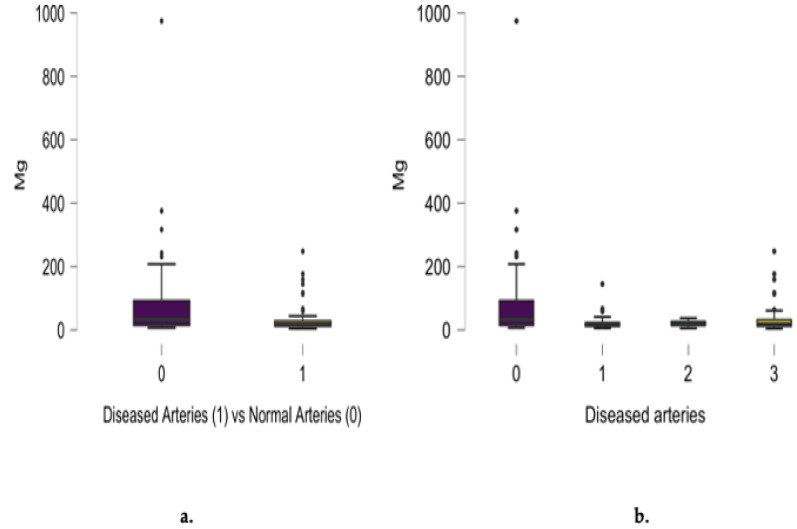
Differences between diseased and normal angiograms regarding scalp hair magnesium concentration (mg/kg) (**a**) and the inverse correlation between angiographic results and the number of diseased coronary arteries (r = −0.237, *p* = 0.003) (**b**).

**Figure 7:**
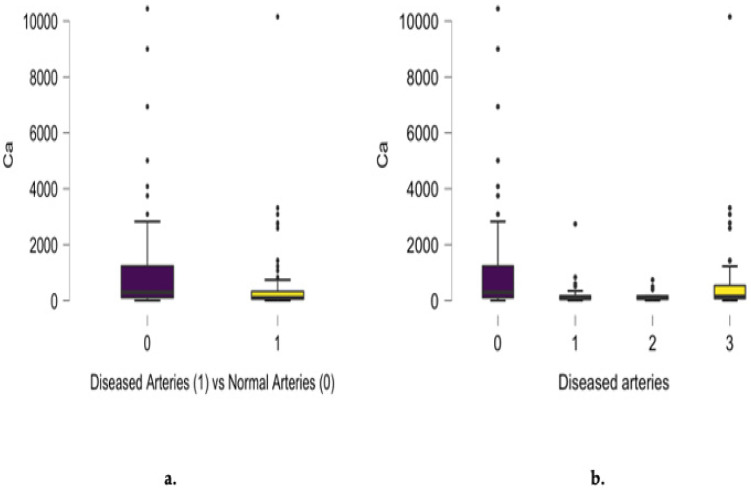
Differences between diseased and normal angiograms regarding scalp hair calcium concentration (mg/kg) (**a**) and the inverse correlation between angiographic results and the number of diseased coronary arteries (r = −0.217, *p* = 0.007) (**b**).

An inverse correlation was noticed between coronary artery disease advancement and magnesium (r = −0.237, *p* = 0.003) and calcium (r = −0.217, *p* = 0.007) concentration, as presented in Figure 6 and Figure 7.

Statistically significant differences between diseased and normal angiograms regarding scalp hair magnesium concentration were noted (*p* = 0.012), as presented in Figure 6a. A significant difference between magnesium concentration and the number of involved arteries was noted for single-, two- and three-vessel disease. Analyzing magnesium hair content in epicardial disease subgroups, higher levels were noted in three-vessel-disease patients. When comparing the patients with normal angiograms and the three-vessel disease patients, their magnesium hair concentrations were found to be significantly different (*p* = 0.0046). An inverse correlation in the entire group between magnesium concentration and the number of diseased coronary arteries was confirmed (r = −0.237, *p* = 0.003), as presented in Figure 6b. 

Statistically significant differences between diseased and normal angiograms regarding scalp hair calcium concentration were found (*p* = 0.007), as presented in Figure 7a. A significant difference between hair calcium content and the number of involved arteries was noted for single-, two- and three-vessel disease. Analyzing calcium hair concentration in epicardial disease subgroups, higher levels were noted in three-vessel disease patients. When comparing the patients with normal angiograms and three-vessel disease patients, their calcium hair concentrations were found to be significantly different (*p* = 0.0040). An inverse correlation in the entire group between calcium concentration and the number of diseased coronary arteries was confirmed (r = −0.217, *p* = 0.007), as presented in Figure 7b.

## 4. Discussion

The unique value of this study’s analysis is based on the correlation between the scalp hair trace element content and coronary artery disease advancement. Our results show a relatively low but significant correlation between the trace element concentration measured in the scalp hair and the number of diseased coronary arteries. To the best of our knowledge, this is the first study that relates the advancement of coronary disease to body trace element content.

Since a significant correlation with one- and two-vessel but not with three-vessel disease was noted, it might be considered an “all or nothing” phenomenon. The presented analysis is hypothesis-generating, and further studies are necessary to corroborate the results from a clinical perspective.

The trace elements are co-factors of various enzymes and are claimed to be involved in different stages of atherosclerosis plaque formation [16]. There are well-known risk factors for coronary artery disease occurrence, but not for its expansion. As there is no explanation for the different levels of advancement of coronary disease, the body concentration of trace elements may be one of the possible indicators of process activation that leads to the formation of atherosclerotic lesions. 

The results of our study indicate the possible relationship between scalp hair sodium concentration and coronary disease extension. Ma et al. [17] revealed the relationship between sodium intake and cardiovascular risk in their large cohort study. Their analysis aimed to estimate the risk of acute syndromes, contrary to our study based on coronary disease extension, including multivessel involvement. Nordlohne et al. [18] suggested a possible link between sodium and interleukin (IL)-17A released by innate and adaptive leukocytes, activating macrophages and T-cell pathways in experimental atherosclerosis. We wish to express the possible role of reducing the content of sodium in daily diets to prevent coronary artery disease development.

Our analysis presented a possible relationship between ultra-trace redox-sensitive elements such as vanadium and coronary disease. In patients presenting with anginal symptoms that were either angiographically proven or involved normal epicardial arteries, we found significant differences between those with different vanadium scalp hair concentrations. A positive relationship between vanadium concentration and the number of involved arteries was noted for single-, two-, and three-vessel disease. The highest differences in vanadium scalp hair concentration were noted between patients presenting with normal angiograms and those with single-vessel disease. Vanadium is environmentally distributed by industrial activity in the atmosphere, soil, water bodies, and sediments and is considered highly toxic [19]. In experimental studies [20], oxidative stress activation in animal hearts due to the toxic properties of excessive vanadium accumulation was noted. In accordance with the results of our analysis, both exposure to and supplementation of vanadium should be avoided.

Chromium naturally exists in small amounts in plants, animals, and the environment and is considered one of the heavy metals that may promote reactive oxygen species production, which induces inflammation, resulting in endothelial dysfunction and an increased risk of atherosclerosis [21]. According to our analysis, chromium was one of the trace elements positively correlated with coronary artery disease. Differences between diseased and normal angiograms regarding scalp hair chromium concentration were noted in this study. A positive relationship between chromium concentration and the number of involved arteries was noted for single- and two-vessel disease. Our previous analysis found a significantly higher chromium concentration in scalp hair samples from carotid artery patients than in those from the control group [14]. Chromium stimulates the synthesis of fatty acids and is responsible for the transport of amino acids into cells. It helps to balance good cholesterol (HDL) and bad cholesterol (LDL), which may prevent atherosclerosis [22]. The results of our analysis indicate that food products that are characterized by an increased chromium content should be avoided to lower the coronary artery disease risk.

Previous reports presented the relationship between urinary nickel concentration and cardiovascular risk [23]. The nickel results obtained from our scalp hair analysis confirm the relationship between nickel concentration and the advancement of coronary disease. This heavy metal is considered a pollutant of food, drinking water, and soil due to industrial environmental contamination. As nickel toxicity has become a global health concern due to its widespread industrial applications, the awareness of its toxicological effects on the immune system has recently increased [24]. Nickel is believed to exert cardiotoxic effects by generating free radicals and increasing lipoperoxidation [25]. A Swedish cross-sectional study presented the relationship between the number of atherosclerotic lesions in carotid arteries and exposure to nickel [26]. In our results, though non-significant differences between diseased and normal angiograms regarding scalp hair nickel concentration were noted, a positive relationship between nickel concentration and the number of involved arteries was noted for single- and two-vessel disease. From a clinical point of view, our analysis may indicate that nickel should be removed from nickel-containing household objects, rechargeable batteries, cell phones, and cigarette lighters to reduce contamination.

Our analysis revealed the inverse correlation between the advancement of coronary artery disease and magnesium concentration. We found statistically significant differences between diseased and normal angiograms regarding scalp hair magnesium concentration and an inverse correlation between magnesium content and the number of diseased coronary arteries. Analyzing the magnesium content in our group presenting with epicardial disease, higher levels were noted in three-vessel disease patients, but these levels were still significantly lower than in patients with normal angiograms. Magnesium may potentially influence cardiovascular disease pathogenesis, as its role in maintaining normal cellular physiology and metabolism is well established [27]. It acts as a cofactor of numerous enzymes involved in the proliferation and migration of endothelial and vascular smooth muscle cells. Rooney et al. [28] presented the relationship between a low magnesium serum concentration and an increased risk for coronary artery disease in their meta-analysis. Ye et al. [29] also confirmed the role of magnesium depletion in an elevated risk of all-cause cardiovascular mortality. A genetic predisposition to hypomagnesemia and increased cardiovascular morbidity was presented in a Mendelian study [30]. According to our results, as in previous reports, magnesium supplementation can be useful in clinical practice as a possible modulator against coronary atherosclerotic plaque formation.

The role of calcium ions in plaque calcification, followed by arterial lumen mineralization, represents the advanced stage of coronary disease. Statistically significant differences between diseased and normal angiograms regarding scalp hair calcium were found in our analysis, indicating an inverse correlation between calcium content and the number of involved arteries. Analyzing the calcium hair concentration in epicardial disease subgroups, higher levels were noted in three-vessel disease patients, but these levels were still statistically significantly lower than in patients with normal angiograms. This process is mainly driven by inflammatory cells that result in the osteoblast differentiation of vascular smooth muscle cells [31]. Our analysis revealed the inverse relationship between calcium concentration and the number of diseased arteries in angiograms. From a clinical perspective related to calcium supplementation, Sim et al.’s [32] meta-analysis did not support any beneficial or adverse effects on cardiovascular morbidity. Cormick et al. [33] suggested the possible indirect role of calcium supplementation on CV risk through blood pressure normalization. The performed analysis may be regarded as a possible indicator for calcium supplementation against epicardial plaque development.

### Study Limitation

This single-center study was performed on a white Caucasian population who presented with standard dietary habits. Vegetarians were not included in the analysis.

## 5. Conclusions

The chronic inflammatory background of coronary artery disease may result from the activation of various enzymatic processes that result in atherosclerotic lesion topography. The number of epicardial arteries involved in patients may result from inflammatory courses that vary between the patients and their co-factors, as trace elements could be regarded as possible indicators. The positive correlation between the number of diseased coronary arteries and sodium, vanadium, chromium, and nickel concentration and its inverse correlation with magnesium and calcium was noted in the performed analysis, suggesting differences in the pathophysiology of lesion formation between patients with diverse levels of advancement of epicardial disease. Since a significant correlation with one- and two-vessel but not with three-vessel disease was noted, it might be considered an “all or nothing” phenomenon. The presented analysis is hypothesis-generating, and further studies are necessary to corroborate the results from a clinical perspective.

## Figures and Tables

**Table 1:** Patients’ characteristics.

Parameters	Analyzed Group (*n* = 157)	Patients with Coronary Artery Disease in Angiograms Group 1 (*n* = 83)	Patients with Normal Angiograms Group 2 (*n* = 74)	*p* (Group 1 vs. 2)
Demographics:				
Age (years) (median (Q1–Q3))	71 (65–75)	71 (65–75)	72 (66–76)	0.375
Sex (male/female)	92/65	57/26	44/30	0.247
Height (cm) (median (Q1–Q3))	168 (160–176)	165 (160–173)	164 (159–174)	0.328
Weight (kg) (median (Q1–Q3))	84 (70–95)	83 (72–95)	85 (68–95)	0.589
CCS class (median (Q1–Q3))	2.0 (1.8–2.2)	2.1 (1.9–2.3)	2.0 (1.7–2.2)	0.867
Co-morbidities:				
Arterial hypertension (*n*, %)	137 (87)	76 (92)	61 (82)	0.088
Hypercholesterolemia (*n*, %)	141 (90)	78 (94)	63 (85)	0.069
Diabetes mellitus (*n*, %)	58 (37)	33 (40)	25 (34)	0.509
Stroke (*n*, %)	13 (8)	7 (8)	6 (8)	0.944
COPD (*n*, %)	10 (6)	4 (5)	6 (8)	0.403
Nicotine (*n*, %)	80 (51)	38 (46)	42 (57)	0.994
Pharmacotherapy:				
B–Blocker (*n*, %)	133 (85)	73 (88)	60 (82)	0.235
ACE-I (*n*, %)	92 (59)	57 (69)	35 (48)	0.007
ARB (*n*, %)	38 (24)	16 (19)	22 (30)	0.129
Flozins (*n*, %)	33 (21)	25 (12)	8 (11)	0.003
Diuretics (*n*, %)	86 (55)	51 (61)	35 (48)	0.077
CCB (*n*, %)	62 (39)	35 (42)	27 (37)	0.470
MRA (*n*, %)	53 (34)	34 (41)	19 (26)	0.044
ARNI (*n*, %)	3 (19)	2 (24)	1 (1)	0.635
Statins (*n*, %)	147 (94)	82 (99)	65 (89)	0.005
ASA (*n*, %)	121(77)	75 (90)	46 (63)	<0.001
Echocardiography:				
Left ventricular ejection fraction (%) (median (Q1–Q3))	63 (60–65)	62 (58–66)	63 (61–66)	0.849
Laboratory results:				
Creatinine (umol/L) (median (Q1–Q3))	84 (74–97)	87 (77–105)	82 (73–93)	0.068
WBC (10^9^/L) (median (Q1–Q3))	7.1 (6.0–8.9)	7.5 (6.3–9.0)	6.9 (5.7–8.6)	0.105
Hemoglobin (kg) (median (Q1–Q3))	8.6 (8.0–9.2)	8.8 (8.1–9.1)	8.5 (8.0–9.1)	0.318
Haematocrit (%) (median (Q1–Q3))	41 (38–44)	42 (39–45)	41 (38–45)	0.201
Platelets (10^3^/L) (median (Q1–Q3))	214 (177–253)	217 (175–249)	213 (180–252)	0.854
Total cholesterol (mmol/L) (median (Q1–Q3))	3.8 (3.3–4.8)	3.7 (3.2–4.7)	4.0 (3.4–5.0)	0.138
HDL fraction (mmol/L) (median (Q1–Q3))	1.2 (1.0–1.5)	1.2 (1.0–1.4)	1.2 (1.1–1.6)	0.039
LDL fraction (mmol/L) (median (Q1–Q3))	2.2 (1.5–2.9)	1.9 (1.4–2.9)	2.3 (1.7–2.8)	0.148
TG (mmol/L) (median (Q1–Q3))	1.3 (1.0–1.7)	1.3 (1.1–1.7)	1.3 (1.0–1.7)	0.452
Glucose (mg/dL) (median (Q1–Q3))	6.0 (5.4–7.3)	6.2 (5.5–8.0)	5.9 (5.4–6.8)	0.310
Uric acid (umol/L) (median (Q1–Q3))	342 (297–407)	347 (306–407)	328 (290–405)	0.316

Abbreviations: ACE-I—angiotensin-converting enzyme inhibitors; ARB—angiotensin receptor blocker; ARNI—angiotensin receptor-neprilysin inhibitor; ASA—aspirin; B-Blockers—beta blockers; CCB—calcium channel blocker; CCS—Canadian Cardiovascular Society; COPD—chronic obstructive pulmonary disease; HDL—high-density lipoprotein; LDL—low-density lipoprotein; Q—quartile; TG—triglycerides; WBC—white blood count.

**Table 2:** Scalp hair trace element concentrations.

Scalp Hair Trace Elements (mg/kg)	Analyzed Group	Patients with Coronary Artery Disease in Angiograms Group 1 (*n* = 83)	Patients with Normal Angiograms Group 2 (*n* = 74)	*p* (Group 1 vs. 2)
Li (median (Q1–Q3))	0.02 (0.01–0.05)	0.02 (0.01–0.05)	0.02 (0.01 = 0.05)	0.682
Na (kg) (median (Q1–Q3))	241 (164–388)	287 (198–529)	199 (151–294)	<0.001
Mg (kg) (median (Q1–Q3))	21.1 (12.2–42.0)	16.9 (11.2–28.2)	31.8 (13.8–92.4)	<0.001
Ca (kg) (median (Q1–Q3))	100 (70–708)	100 (54–330)	295 (103–1239)	<0.001
V (kg) (median (Q1–Q3))	0.02 (0.00–0.03)	0.03 (0.00–0.03)	0.02 (0.00–0.03)	0.005
Cr (kg) (median (Q1–Q3))	0.94 (0.62–1.43)	1.04 (0.75–1.73)	0.75 (0.55–1.17)	0.626
Mn (kg) (median (Q1–Q3))	0.20 (0.13–0.31)	0.20 (0.14–0.30)	0.22 (0.14–0.34)	<0.001
Fe (kg) (median (Q1–Q3))	10.7 (8.7–15.1)	11.8 (9.3–16.4)	10.1 (8.7–13.0)	0.023
Co (kg) (median (Q1–Q3))	0.02 (0.01–0.04)	0.02 (0.01–0.03)	0.02 (0.01–0.05)	0.333
Ni (kg) (median (Q1–Q3))	0.78 (0.57–1.22)	0.87 (0.68–1.39)	0.66 (0.53–1.09)	0.014
Zn (kg) (median (Q1–Q3))	152 (119–171)	152 (117–170)	153 (125–171)	0.777
Cu (kg) (median (Q1–Q3))	13.3 (10.8–19.6)	12.3 (10.5–16.7)	14.4 (10.9–24.1)	0.038

Abbreviations: Ca—calcium; Co—cobalt; Cr—chromium; Cu—copper; Fe—iron; kg—kilogram, Li—lithium; mg—milligram, Mg—magnesium; Mn—manganese; *n*—number, Ni—nickel; Na—sodium; Q—aurtile, V—vanadium; Zn—zinc.

## Data Availability

The created data and analysis will be available for three years following the publication of this article upon reasonable request to the corresponding author.
